# Mental Health of Elementary Schoolteachers in Southern Brazil: Working Conditions and Health Consequences

**DOI:** 10.1155/2015/825925

**Published:** 2015-08-23

**Authors:** Marta Regina Cezar-Vaz, Clarice Alves Bonow, Marlise Capa Verde de Almeida, Laurelize Pereira Rocha, Anelise Miritz Borges

**Affiliations:** ^1^School of Nursing, Federal University of Rio Grande, 96203-900 Rio Grande, RS, Brazil; ^2^Federal University of Pampa, Uruguaiana, RS, Brazil

## Abstract

The mental health of educators is a growing problem in many countries. This study sought to identify self-reported stressful working conditions of elementary schoolteachers and the biopsychosocial consequences of those working conditions and then identify working conditions that promote well-being for teachers in the workplace. Exploratory study was done with 37 teachers. Data collection was performed using a structured interview with a questionnaire. Results show that stressful working conditions are related to inadequate salary, an excessive number of activities, and having to take work home. Biopsychosocial consequences include anxiety, stress, and sleep disorders. There was a statistically significant association between inadequate salary and anxiety (*p* = 0.01) and between an excessive number of activities and stress (*p* = 0.01). Teachers reported that a good relationship among colleagues is a working condition that promotes well-being in the workplace. The identification of stressful working conditions for teachers, the biopsychosocial consequences, and working conditions that promote well-being in the workplace are relevant to determining actions that improve the work environment and, consequently, the health of teachers.

## 1. Introduction

This paper discusses stressful working conditions of elementary schoolteachers and the elementary schoolteachers' self-reported biopsychosocial consequences due to stressful working conditions. It also presents working conditions that generate well-being for teachers at school.

Studies evidence the existence of stressful working conditions in different professions. In South Africa, a study with construction workers showed that tight deadlines and long working hours contributed to making work more stressful [1]. In Uganda [2] and China [3], nurses are targets of stressful working conditions. In Brazil, a study conducted with bank workers indicates that the exposure to adverse psychosocial factors at work is an independent marker of self-reported poor health [4, 5]. A study with policewomen also showed that work-related stress may have an impact on family life [6].

In recent years, stressful working conditions for teachers have increasingly become a problem in many countries [7–10]. The World Health Organization along with UNESCO promotes principles of quality teaching by means of a Recommendation Concerning the Status of Teachers [11]. This Recommendation provides a definition of teachers' responsibilities and rights in the workplace and sets guidelines for a dialogue among educational authorities, teachers, and their respective associations.

This concern arises from the fact that teaching is acknowledged as a strenuous activity [7–10, 12, 13]. The stressful conditions faced in a teacher's daily routine may lead to an imbalance between work and physical and mental health, resulting in the development of stress [12, 13]. A study carried out with teachers in the United States found that a lack of resources and being overworked are sources of stress [9]. In a study conducted in Finland, stressful conditions in the workplace are directly related to an overload of tasks, in addition to classes, an excessive number of students per class, a heavy workload, the need for teachers to have more than one job, and a low monthly income [14]. Furthermore, a lack of social and economic stability increases the risk of occupational burnout in all age groups [7]. Conflicting roles, the loss of control and/or autonomy, and a lack of social support are the most important predisposing factors [15]. In studies conducted in Brazil, teachers point to stress indicators such as headaches, drowsiness and heart palpitation, results of inadequate wages, an excessive workload, and multiple responsibilities [16–18].

Therefore, this study sought to identify self-reported stressful working conditions of elementary schoolteachers and the biopsychosocial consequences of those working conditions and then identify working conditions that promote well-being for teachers in the workplace.

## 2. Materials and Methods

Exploratory and descriptive study with elementary schoolteachers was conducted in 2012 in a small city (39,685 inhabitants) [19] in Southern Brazil. This study is part of a larger research project titled “Health, Risks and Occupational Diseases: An Integrated Study in Different Work Environments” [20]. The Institutional Review Board at the Federal University of Rio Grande (Universidade Federal do Rio Grande (FURG)) approved the study. The elementary schoolteachers participating in the study signed free and informed consent forms. The study was linked to the Laboratory of Socio-Environmental Process Studies and Collective Health Promotion (LAMSA) research group, Nursing School, Federal University of Rio Grande.

### 2.1. Subjects

The study subjects were elementary schoolteachers from a city in the metropolitan region of Porto Alegre, State of Rio Grande do Sul, Brazil. The city has 27 state schools and 236 elementary schoolteachers. All teachers were invited to participate in the research. Only 16% of the elementary schoolteachers' population participated; this means that thirty-seven teachers in 21 public elementary schools participated, characterizing sample convenience. Although this is not a representative sample of the population, it presents tendency of stressful working conditions and the biopsychosocial consequences for the health of elementary schoolteachers.

Table 1 shows the demographic and occupational characteristics of the participants. All participants were female; most were between 30 and 40 years of age (41.8%), were married (73%), have obtained a postgraduate degree (45.9%), have worked at one school (75.7%) for over 10 years (51.4%), and spend 30–40 hours per week with their students (37.8%).

### 2.2. Questionnaire and Data Collection

Data collection was performed in June 2012, using a structured interview with a questionnaire including multiple-choice and single-choice questions. The questionnaire was developed by the LAMSA team based on the theoretical foundation of the International Labour Organization [11] on working conditions as determinants of occupational health. Moreover, the choice of topics was based on the interests of the group of teachers as expressed to the research coordinator at the advanced university campus. All study participants completed the questions within the 45 minutes allocated to answer the questionnaire.

The questionnaire included multiple-choice and single-choice questions with the following variables: participant characteristics (gender, age, skin color/ethnic background, educational level, and marital status); occupational data (number of schools they work at, length of time at the school, length of time working as a teacher, number of hours per day spent with the students, and salary income); stressful working conditions (room size inadequate for the number of students, inadequate or insufficient working material, noisy school, daily dissension, tension and insecurity, need to take work home, inadequate salary, difficulty maintaining the students concentration in the classroom, lack of recognition, excessive activities, excessive workload, high number of students per classroom, school demands, demands of society in relation to the education of students, and students' lack of respect); biopsychosocial consequences (use of anxiolytics, physical activity, isolation, irritability, headaches, increased use of cigarettes, anxiety, depression episodes, panic attacks, stress, disorders of the sleep-wake cycle, waking up several times during the night, waking up tired in the morning, and sleepiness); and conditions that promote well-being for teachers in the workplace (harmony among colleagues, working with respect, rapport among colleagues, and autonomy in the workplace).

### 2.3. Data Analysis

Simple frequency distribution (absolute numbers and percentages) was used to describe the demographic and occupational characteristics and the frequency of stressful working conditions, health consequences for teachers, and working conditions that promote well-being for teachers in the workplace. Pearson correlation analysis was performed to examine the associations between the five major stressful working conditions and the five major health consequences for teachers. The Statistical Package for Social Sciences (SPSS) software, Version 19.0, was used.

## 3. Results

The five stressful working conditions listed by elementary schoolteachers include inadequate salary (56.8%), inadequate or insufficient work material (54.1%), size of the classroom being inadequate for the number of students (50%), excessive activities (35.1%), and taking work home (32.4%). Other working conditions mentioned by respondents address the difficulty keeping students focused in the classroom, lack of recognition of the profession, noise in the school, excessive workload, school and social demands, and difficulties getting along with colleagues (Table 2).

Regarding the biopsychosocial consequences (Table 3), the five most frequent biopsychosocial consequences to the health of elementary schoolteachers were related to working conditions including anxiety (78.4%), headache (59.5%), stress (54.1%), waking up several times during the night (51.4%), and irritability (48.6%). Other reactions mentioned by the respondents include difficulty in sleeping at night, morning tiredness, use of calming medication, depression, and isolation.

From Pearson correlation, the five most frequent stressful working conditions and health consequences for elementary schoolteachers were examined. There was a statistically significant correlation between two variables (Table 4). There was a positive correlation between self-reported stressful working conditions and receiving an inadequate salary with stress (*p* = 0.01) and between excessive activities and self-reported health consequence for teachers with anxiety (*p* = 0.01).

Elementary schoolteachers also reported working conditions that promote well-being at school (Table 5). Teachers indicated that working relationships that promote rapport (73%), respect (67.6%), and harmony (59.5%) favor well-being.

## 4. Discussion

The results of this study show that major stressful working conditions for elementary schoolteachers in Southern Brazil involve inadequate salary, lack of structure at school (material and physical), excessive activities, and having to take work home. These results are similar to those found in the literature and are related to the biopsychosocial consequences for the health of teachers, such as anxiety, headaches, stress, inadequate sleep, and irritability [7–10].

The inadequate salary of teachers in Finland, the main self-reported stressful condition of work by the teachers in this study, was associated with stress and the development of other mental disorders [14]. In a study carried out in China [10], it was associated with turnover rates for teachers, with a high level of stress, inadequate breaks and holidays, heavy workload, and negative student behavior. Furthermore, a study in Pakistan [21] with a group of 1,020 students and 204 high schoolteachers indicated that the poor socioeconomic status (low salaries) of teachers affects their performance.

Another important stressful working condition for elementary schoolteachers who participated in this study was the lack of structure at the workplace (inadequate or insufficient work material). A study in Greece [22] pointed out that work-related stress is related to poor working conditions, such as lack of teaching materials. Elementary schoolteachers identified the size of the classroom as inadequate for the number of students. This situation was also observed in South Africa [23]; however the educational transformation of that country forced the managers, by means of teacher's qualification and adjustments made in schools, and teachers to improve the school environment and, therefore, student learning.

Teachers in Spain identified an excessive number of activities at work as also being a stressful condition, in a study evaluating the association between psychiatric morbidity and working conditions. The study showed that psychiatric morbidity was associated with excessive activities [24].

A study designed to evaluate the workload of teachers in Germany and their mental health showed that teachers work more than 51 hours per week [7] and that it is also necessary for them to take work home.

The biopsychosocial consequences for the health of teachers related to stressful working conditions involved anxiety, headaches, stress, waking up several times during the night, and irritability. A study in Hong Kong [25] with 1,710 primary or secondary schoolteachers highlighted anxiety, headaches, and sleep problems among the ten most frequent health complaints. Other complaints included tiredness, eyestrain, voice disorders, shoulder pain, neck pain, cold/flu, and lower-back pain.

Stress was evaluated in a study of 203 primary schoolteachers in Taiwan [26]. Twenty-six percent of the teachers reported that being a teacher was either very or extremely stressful. The most effective coping strategy reported was having a healthy family life. Teachers reported that the most effective action that schools or the government could take to reduce their stress was to decrease their workload. Furthermore, it is known that stress can manifest as irritability. And irritability may occur in the workplace, which is the classroom for teachers, or at home [27].

However, the school environment is not only made of stressful conditions. Teachers reported that rapport among colleagues, respect, harmonious coexistence, and autonomy in the workplace help minimize stressful conditions and thus transform the school into a healthier environment. This is similar to recommendations found in a study conducted in Malaysia regarding the stress of elementary schoolteachers. The study recommends that the school environment promotes good relationships among colleagues and provides adequate resources and facilities to minimize stressful work conditions [8].

*Limitations*. One limitation was the number of teachers who participated in the study and also using sample of a small village in the South of Brazil is a limitation, causing problems to extrapolate results to other bigger cities or to other countries. However, stressful working conditions of elementary schoolteachers are a public health problem evidenced on other Brazilian cities [16, 17] and on other countries, as Germany [7] and Malaysia [8], with similar population. And the findings corroborate evidence provided elsewhere in the literature regarding stressful conditions caused by inadequate wages [10, 14, 21], intense weekly workload [10], and an excessive number of activities [24], which can generate biopsychosocial consequences such as anxiety, irritability at home or school, headaches, and sleep disorders [25, 27].

Another limitation is the lack of research in regard to association between stressful working conditions and conditions unrelated to work, such as family support [26]. However, one study shows evidences that an adequate relationship among colleagues is a work condition that favors the well-being of teachers in the school environment [8]. Still, it presents the limitation of the data analysis method which has a tendency to correlate stressful working conditions and the biopsychosocial consequences for the health of elementary schoolteachers.

Besides these, another important limitation is the lack of male subjects. This is an important factor because it is necessary to consider the prevalence of anxiety disorders, which indeed are more frequent in female persons. This was evident in study within 250 college teachers [28] where results indicated significant differences between male and female college teachers on anxiety, showing women to be more anxious.

## 5. Conclusions

From the results obtained, it was observed that stressful working conditions are related to the consequences for the health of elementary schoolteachers. This profession requires attention due to the different work-related biopsychosocial consequences.

Results show correlations between inadequate salary and anxiety and between number of activities and stress. These correlations show that there needs to be a change in teachers' working conditions to produce healthy conditions at work.

Investigation by health professionals, anywhere in the world, contributes to planning interventions in this field, improving the work environment and, consequently, the health of teachers. Additionally, the implementation of policies designed to increase the recognition of these professionals by principals, students, and parents, especially in state schools, could help protect the mental health of teachers and contribute to their professional performance and health. This is justified because recognition is not a factor analyzed in the top five of stressful working conditions listed by elementary schoolteachers. The factor lack of recognition was located seventh on the working condition list.

## Figures and Tables

**Table 1:** Demographic and occupational characteristics^*∗*^.

Characteristic	*n*	Percentage
Age (years)		
19–29	5	13.8
30–40	15	41.8
41–50	11	30.6
>50	5	13.8

Marital status		
Single	7	18.9
Married	27	73.0
Divorced	3	8.1

Highest educational level attained		
Secondary school	4	10.8
Higher education, incomplete	2	5.4
Higher education	7	18.9
Postgraduation, incomplete	7	18.9
Postgraduation	17	45.9

Skin color/ethnic background		
White	34	91.9
Brown	2	5.4

How many schools do you work at?		
One	28	75.7
Two	7	18.9
Three	2	2.6

Time working at the school		
<1 year	3	8.1
1–5 years	10	27.0
5–10 years	5	13.5
>10 years	19	51.4

Time working as a teacher		
<1 years	1	2.8
1–5 years	6	16.7
5–10 years	6	16.7
>10 years	23	63.9

Hours spent with students per week		
<10 hours	5	13.5
10–20 hours	8	21.6
20–30 hours	3	8.1
30–40 hours	14	37.8
>40 hours	3	8.1

Monthly income		
Up to U.S. $419	2	5.7
U.S. $419–U.S. $838	23	65.7
U.S. $838–U.S. $1.047	10	28.6

^*∗*^Numbers for each item may be less than the total numbers because of missing values.

**Table 2:** Frequency of stressful working conditions in the school as self-reported by elementary schoolteachers.

Working conditions	*n*	Percentage
Inadequate salary	**21**	**56.8**
Inadequate or insufficient work material	**20**	**54.1**
Size of the classroom inadequate for the number of students	**18**	**50.0**
Excessive activities	**13**	**35.1**
Taking work home	**12**	**32.4**
Difficulty maintaining the concentration of students in the classroom	11	29.7
Lack of recognition	11	29.7
Noisy school	7	18.9
Excessive workload	7	18.9
Society demands in relation to the education of students	5	13.5
Daily dissension between colleagues	2	5.4
School demands	2	5.4
Difficult interactions with colleagues	1	2.7
Tension and insecurity in the workplace	1	2.7

**Table 3:** Frequency of biopsychosocial consequences for the health of elementary schoolteachers related to stressful working conditions.

Health consequences	*n*	Percentage
Anxiety	**29**	**78.4**
Headache	**22**	**59.5**
Stress	**20**	**54.1**
Waking up several times during the night	**19**	**51.4**
Irritability	**18**	**48.6**
Waking up tired in the morning	10	27.0
Circadian rhythm sleep disorder	8	21.6
Drowsiness	8	21.6
Use of anxiolytics	7	18.9
Depression episodes	7	18.9
Insomnia	7	18.9
Isolation	4	10.8
Increase in cigarette use	1	2.7
Panic disorder	1	2.7

**Table 4:** Correlation between the main self-reported working conditions and biopsychosocial consequences for the health of teachers.

Stressful working conditions	Anxiety		Headache		Stress		Waking up several times during the night		Irritability	
	*ρ*	*p* value	*ρ*	*p* value	*ρ*	*p* value	*ρ*	*p* value	*ρ*	*p* value
Inadequate salary	−0.06	0.72	−0.16	0.32	**0.39**	**0.01**	0.13	0.43	−0.02	0.89
Inadequate or insufficient work material	−0.19	0.26	−0.16	0.33	−0.22	0.18	−0.04	0.78	0.04	0.78
Size of the classroom inadequate for the number of students	0.12	0.46	0.06	0.71	0.21	0.19	−0.25	0.13	0.01	0.91
Excessive activities	**0.38**	**0.01**	0.03	0.85	0.22	0.18	−0.19	0.26	0.19	0.26
Taking work home	0.08	0.63	0.12	0.46	−0.19	0.24	0.15	0.35	0.17	0.30

**Table 5:** Working conditions that promote well-being for teachers at school.

Healthy conditions	*n*	Percent
Rapport among colleagues	27	73.0
Respect among colleagues	25	67.6
Harmonious coexistence with colleagues	22	59.5
Autonomy in the workplace	15	40.5

## References

[1] Bowen P., Edwards P., Lingard H. (2013). Workplace stress experienced by construction professionals in South Africa. *Journal of Construction Engineering and Management*.

[2] Nabirye R. C., Brown K. C., Pryor E. R., Maples E. H. (2011). Occupational stress, job satisfaction and job performance among hospital nurses in Kampala, Uganda. *Journal of Nursing Management*.

[3] Wu H., Ge C. X., Sun W., Wang J. N., Wang L. (2011). Depressive symptoms and occupational stress among Chinese female nurses: the mediating effects of social support and rational coping. *Research in Nursing and Health*.

[4] Silva L. S., Barreto S. M. (2012). Stressful working conditions and poor self-rated health among financial services employees. *Revista de Saúde Pública*.

[5] Silva L. S., Barreto S. M. (2010). Adverse psychosocial working conditions and minor psychiatric disorders among bank workers. *BMC Public Health*.

[6] Bezerra C. M., Minayo M. C. S., Constantino P. (2013). Estresse ocupacional em mulheres policiais. *Ciência & Saúde Coletiva*.

[7] Bauer J., Unterbrink T., Hack A. (2007). Working conditions, adverse events and mental health problems in a sample of 949 German teachers. *International Archives of Occupational and Environmental Health*.

[8] Abdul Samad N. I., Hashim Z., Moin S., Abdullah H. (2010). Assessment of stress and its risk factors among primary school teachers in the Klang Valley, Malaysia. *Global Journal of Health Science*.

[9] Yang X., Wang L., Ge C., Hu B., Chi T. (2011). Factors associated with occupational strain among Chinese teachers: a cross-sectional study. *Public Health*.

[10] Liu S., Onwuegbuzie A. J. (2012). Chinese teachers' work stress and their turnover intention. *International Journal of Educational Research*.

[11] International Labour Organization. United Nations Educational (2008). *The ILO/UNESCO Recommendation Concerning the Status of Teachers (1966) and the UNESCO Recommendation Concerning the Status of Higher-Education Teaching Personnel (1997) with a User's Guide*.

[12] Shernoff E. S., Mehta T. G., Atkins M. S., Torf R., Spencer J. (2011). A qualitative study of the sources and impact of stress among Urban teachers. *School Mental Health*.

[13] Gold E., Smith A., Hopper I., Herne D., Tansey G., Hulland C. (2010). Mindfulness-based stress reduction (MBSR) for primary school teachers. *Journal of Child and Family Studies*.

[14] Laaksonen E., Martikainen P., Lahelma E. (2007). Socioeconomic circumstances and common mental disorders among Finnish and British public sector employees: evidence from the Helsinki Health Study and the Whitehall II Study. *International Journal of Epidemiology*.

[15] Organização Pan-Americana da Saúde no Brasil (2001). *Doenças relacionadas ao trabalho: manual de procedimentos para os serviços de saúde*.

[16] Costa E. C., Bachion M. M., Godoy L. F., Abreu L. O. (2005). Percepções sobre o estresse entre professores universitários. *Revista da Rede de Enfermagem do Nordeste*.

[17] Neto A. M. S., Araújo T. M., Dutra F. R. D. (2000). Condições de trabalho e saúde de professores da rede particular de ensino de Salvador, Bahia. *Revista Baiana de Saúde Pública*.

[18] Batista J. B. V., Carlotto M. S., Moreira A. M. (2013). Depressão como causa de afastamento do trabalho: um estudo com professores do ensino fundamental. *Psico*.

[19] The Brazilian Institute of Geography and Statistics (IBGE) (2010). *Population Censuses 2010*.

[20] Cezar-Vaz M. R. (2010). *Health, Risks and Occupational Diseases: An Integrated Study in Different Work Environments*.

[21] Nadeem M., Rana M. S., Lone A. H., Maqbool S., Naz K., Ali A. (2011). Teacher's competencies and factors affecting the performance of female teachers in Bahawalpur (Southern Punjab) Pakistan. *International Journal of Business and Social Science*.

[22] Kokkinos C. M. (2007). Job stressors, personality and burnout in primary school teachers. *British Journal of Educational Psychology*.

[23] Msila V. (2008). Transforming teacher practice: a look at the experiences of two first-year teacher-learners in the NPDE programme. *Educational Research and Reviews*.

[24] Moreno-Abril O., Luna del Castillo J. D. D., Fernández-Molina C. (2007). Factors associated with psychiatric morbidity in Spanish schoolteachers. *Occupational Medicine*.

[25] Chong E. Y. L., Chan A. H. S. (2010). Subjective health complaints of teachers from primary and secondary schools in Hong Kong. *International Journal of Occupational Safety and Ergonomics*.

[26] Kyriacou C., Chien P.-Y. (2004). Teacher stress in Taiwanese primary schools. *Journal of Educational Enquiry*.

[27] Independent Education Union (1996). *Education and Stress. Report on the Survey Conducted by the Victoria and NSW/ACT Independent Education Union on Workloads and Perceptions of Occupational Stress among Union Members Employed in Catholic Schools, and Education Offices and in Independent Schools*.

[28] Bedi S., Sehgal M. (2012). Gender differences on occupational stress, ways of coping and anxiety among college teachers. *Indian Journal of Psychological Science*.

